# A guide to selecting high-performing antibodies for VAPB (UniProt ID: O95292) for use in western blot, immunoprecipitation, and immunofluorescence

**DOI:** 10.12688/f1000research.160226.1

**Published:** 2024-12-24

**Authors:** Sara González Bolívar, Riham Ayoubi, Charles Alende, Maryam Fothouhi, Irina Shlaifer, Peter S. McPherson, Carl Laflamme, NeuroSGC/YCharOS/EDDU collaborative group, ABIF consortium

**Affiliations:** 1 Montreal Neurological Institute-Hospital, Montreal, Québec, Canada

**Keywords:** O95292, VAPB, Vesicle-associated membrane protein-associated protein B/C, VAMP-associated protein B/C, antibody characterization, antibody validation, western blot, immunoprecipitation, immunofluorescence

## Abstract

VAPB is an adaptor protein known for its role as an anchor for other proteins at the endoplasmic reticulum. A mutant form of VAPB has been linked to amyotrophic lateral sclerosis and the underlying mechanisms resulting from this defect are studied by researchers in this area to uncover its implication in the disease. Here we have characterized six VAPB commercial antibodies for western blot, immunoprecipitation, and immunofluorescence using a standardized experimental protocol based on comparing read-outs in knockout cell lines and isogenic parental controls. These studies are part of a larger, collaborative initiative seeking to address antibody reproducibility issues by characterizing commercially available antibodies for human proteins and publishing the results openly as a resource for the scientific community. While use of antibodies and protocols vary between laboratories, we encourage readers to use this report as a guide to select the most appropriate antibodies for their specific needs.

## Introduction

VAPB is the vesicle-associated membrane protein (VAMP)-associated protein B and C. ^1^ VAPB acts as a tethering protein of the endoplasmic reticulum to other organelles, orchestrating many cellular processes, namely vesicle trafficking. ^2^ A mutant form of the protein has been linked to amyotrophic lateral sclerosis ^3^ and high-quality antibodies and necessary to explore the resulting pathological mechanisms.

This research is part of a broader collaborative initiative in which academics, funders and commercial antibody manufacturers are working together to address antibody reproducibility issues by characterizing commercial antibodies for human proteins using standardized protocols, and openly sharing the data. ^4– 6^ Here we evaluated the performance of six commercial antibodies for VAPB for use in western blot, immunoprecipitation, and immunofluorescence, enabling biochemical and cellular assessment of VAPB properties and function. The platform for antibody characterization used to carry out this study was endorsed by a committee of industry academic representatives. It consists of identifying human cell lines with adequate target protein expression and the development/contribution of equivalent knockout (KO) cell lines, followed by antibody characterization procedures using most commercially available antibodies against the corresponding protein. The standardized consensus antibody characterization protocols are openly available on Protocol Exchange, a preprint server (DOI: 10.21203/rs.3.pex-2607/v1). ^7^

The authors do not engage in result analysis or offer explicit antibody recommendations. Our primary aim is to deliver top-tier data to the scientific community, grounded in Open Science principles. This empowers experts to interpret the characterization data independently, enabling them to make informed choices regarding the most suitable antibodies for their specific experimental needs. Guidelines on how to interpret antibody characterization data found in this study are featured on the YCharOS gateway. ^8^

## Results and discussion

Our standard protocol involves comparing readouts from WT (wild type) and KO (knockout cells). ^9, 10^ The first step was to identify a cell line(s) that expresses sufficient levels of a given protein to generate a measurable signal using antibodies. To this end, we examined the DepMap (Cancer Dependency Map Portal, RRID:SCR_017655) transcriptomics database to identify all cell lines that express the target at levels greater than 2.5 log _2_ (transcripts per million “TPM” + 1), which we have found to be a suitable cut-off. ^4^ HeLa expresses the VAPB transcript at 5.2 and was identified as a suitable cell line to modify with CRISPR/Cas9 to KO the corresponding *VAPB* gene ( Table 1).

**Table 1.** Summary of the cell lines used.

Institution	Catalog number	RRID (Cellosaurus)	Cell line	Genotype
ATCC	CCL-2	CVCL_0030	HeLa	WT
Montreal Neurological Institute	-	CVCL_B6RU	HeLa	*VAPB* KO

**Figure 1.**
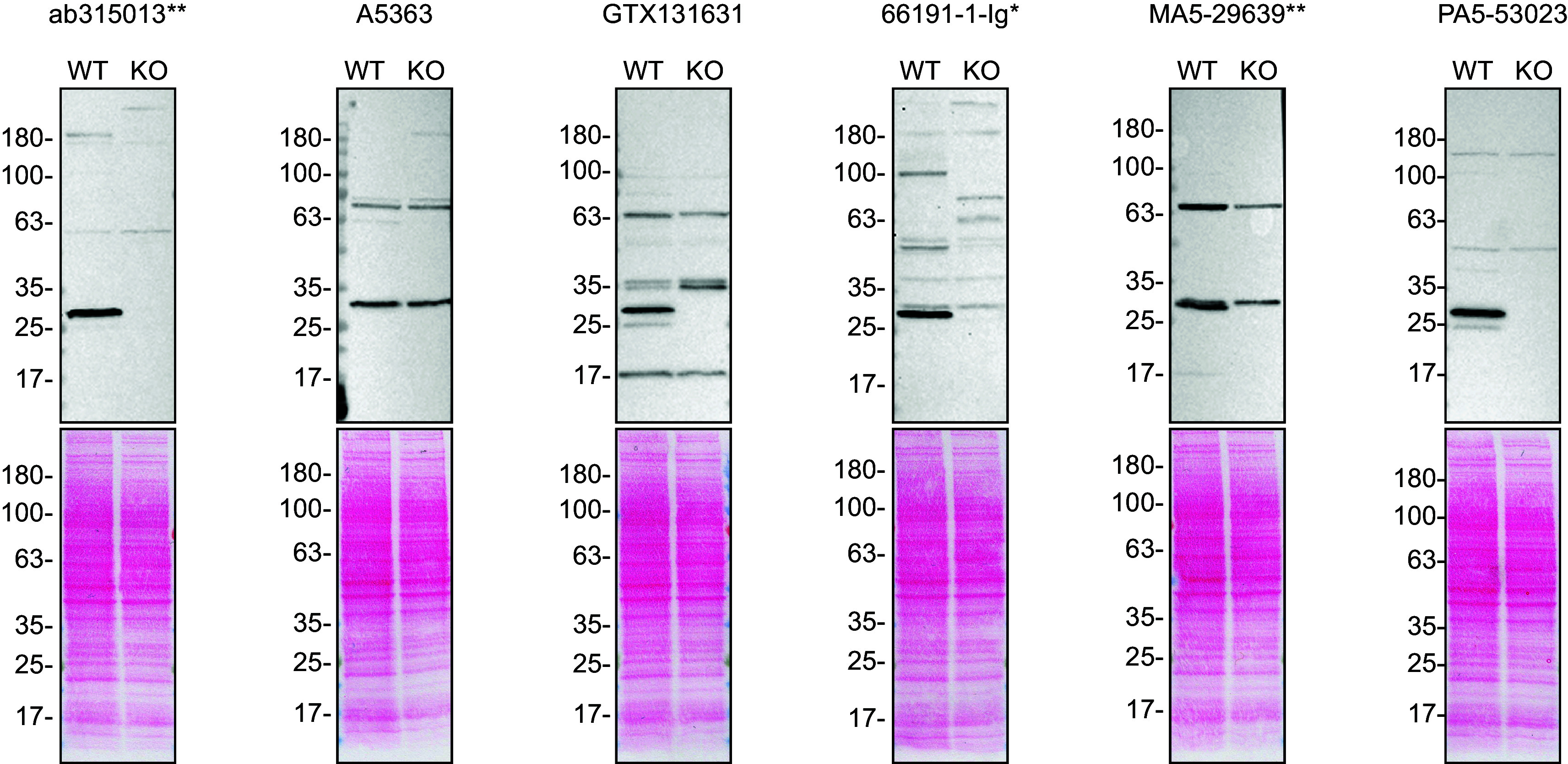
VAPB antibody screening by western blot. Lysates of HeLa WT and *VAPB* KO were prepared, and 40 μg of protein were processed for western blot with the indicated VAPB antibodies. The Ponceau stained transfers of each blot are presented to show equal loading of WT and KO lysates and protein transfer efficiency from the acrylamide gels to the nitrocellulose membrane. Antibody dilutions were chosen according to the recommendations of the antibody supplier. Antibody dilution used: ab315013** at 1/1000, A5363 at 1/500, GTX131631 at 1/1000, 66191-1-Ig* at 1/1000, MA5-29639** at 1/1000, PA5-53023 at 1/1500 (0.2 μg/ml). Predicted band size: 27.2 kDa. **Recombinant antibody, *Monoclonal antibody.

To screen the antibodies by western blot, WT and *VAPB* KO protein lysates were ran on SDS-PAGE, transferred onto nitrocellulose membranes, and then probed with all six VAPB antibodies in parallel ( Figure 1).

**Figure 2.**
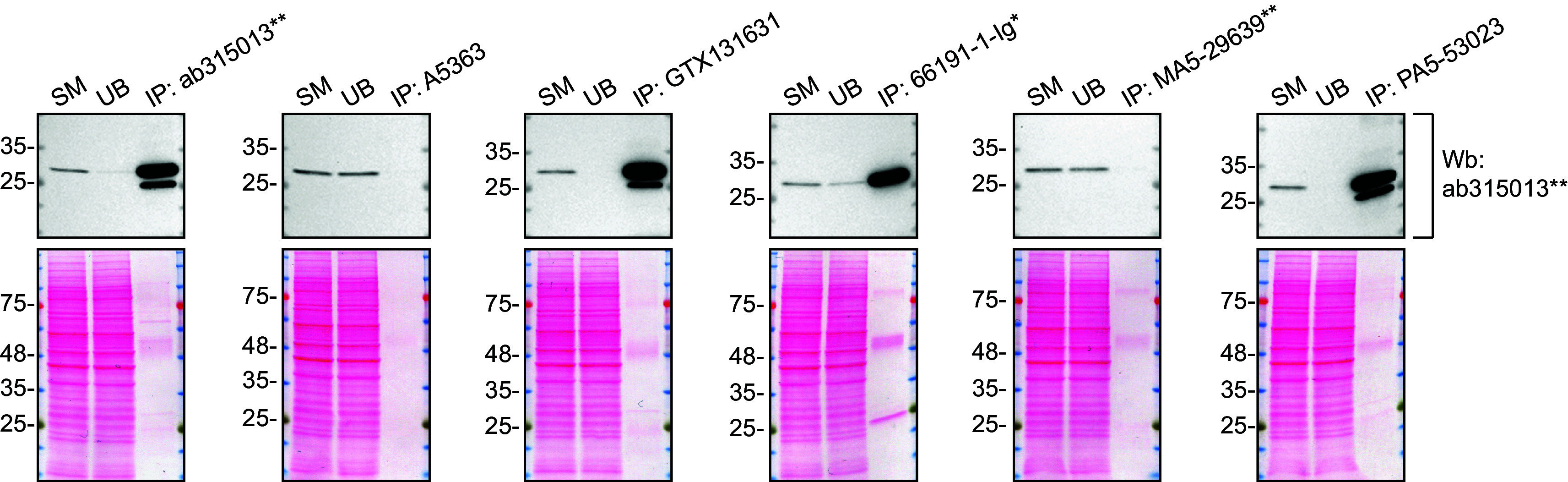
VAPB antibody screening by immunoprecipitation. HeLa lysates were prepared, and immunoprecipitation was performed using 1 mg of lysate and 2.0 μg of the indicated VAPB antibodies pre-coupled to Dynabeads protein A or protein G. Samples were washed and processed for western blot with the indicated VAPB antibody. For western blot, ab315013** was used at 1/1000. The Ponceau stained transfers of each blot are shown. SM=4% starting material; UB=4% unbound fraction; IP=immunoprecipitate. **Recombinant antibody, *Monoclonal antibody.

We then assessed the capability of the six antibodies to capture VAPB from HeLa protein extracts using immunoprecipitation techniques, followed by western blot analysis. For the immunoblotting step, a specific VAPB antibody identified previously (refer to Figure 1) was selected. Equal amounts of the starting material (SM) and the unbound fractions (UB), as well as the whole immunoprecipitate (IP) eluates were separated by SDS-PAGE ( Figure 2).

**Figure 3.**
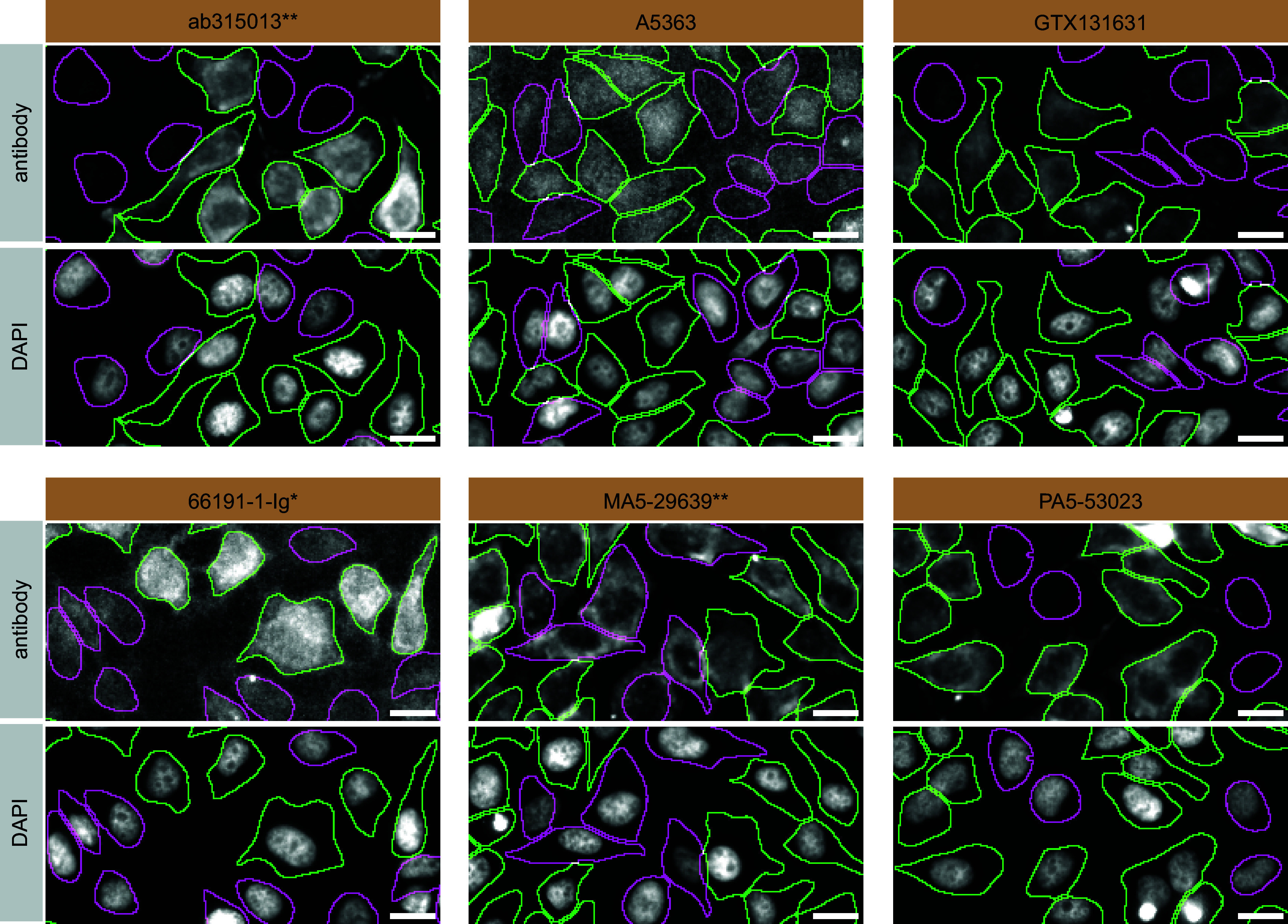
VAPB antibody screening by immunofluorescence. HeLa WT and *VAPB* KO cells were labelled with a green or a far-red fluorescent dye, respectively. WT and KO cells were mixed and plated to a 1:1 ratio on coverslips. Cells were stained with the indicated VAPB antibodies and with the corresponding Alexa-fluor 555 coupled secondary antibody including DAPI. Acquisition of the blue (nucleus-DAPI), green (WT), red (antibody staining) and far-red (KO) channels was performed. Representative images of the merged blue and red (grayscale) channels are shown. WT and KO cells are outlined with green and magenta dashed lines, respectively. When an antibody was recommended for immunofluorescence by the supplier, we tested it at the recommended dilution. The rest of the antibodies were tested at 1 and 2 μg/ml and the final concentration was selected based on the detection range of the microscope used and a quantitative analysis not shown here. Antibody dilution used: ab315013** at 1/250, A5363 at 1/1400, GTX131631 at 1/1000, 66191-1-Ig* at 1/800, MA5-29639** at 1/1000, PA5-53023 at 1/150. Bars = 10 μm. **Recombinant antibody, *Monoclonal antibody.

For immunofluorescence, the six antibodies were screened using a mosaic strategy. First, HeLa WT and *VAPB* KO cells were labelled with different fluorescent dyes in order to distinguish the two cell lines, and the VAPB antibodies were evaluated. Both WT and KO lines imaged in the same field of view to reduce staining, imaging and image analysis bias ( Figure 3). Quantification of immunofluorescence intensity in hundreds of WT and KO cells was performed for each antibody tested, and the images presented in Figure 3 are representative of this analysis. ^7^

In conclusion, we have screened six VAPB commercial antibodies by western blot, immunoprecipitation, and immunofluorescence by comparing the signal produced by the antibodies in human HeLa WT and *VAPB* KO cells. To assist users in interpreting antibody performance, Table 3 outlines various scenarios in which antibodies may perform in all three applications. ^4^ Several high-quality and renewable antibodies that successfully detect VAPB were identified in all applications. Researchers who wish to study VAPB in a different species are encouraged to select high-quality antibodies, based on the results of this study, and investigate the predicted species reactivity of the manufacturer before extending their research.

The underlying data for this study can be found on Zenodo, an open-access repository for which YCharOS has its own collection of antibody characterization reports.

### Limitations

Inherent limitations are associated with the antibody characterization platform used in this study. Firstly, the YCharOS project focuses on renewable (recombinant and monoclonal) antibodies and does not test all commercially available VAPB antibodies. YCharOS partners provide approximately 80% of all renewable antibodies, but some top-cited polyclonal antibodies may not be available through these partners.

Secondly, the YCharOS effort employs a non-biased approach that is agnostic to the protein for which antibodies have been characterized. The aim is to provide objective data on antibody performance without preconceived notions about how antibodies should perform or the molecular weight that should be observed in western blot. As the authors are not experts in VAPB, only a brief overview of the protein’s function and its relevance in disease is provided. VAPB experts are invited to analyze and interpret observed banding patterns in western blots and subcellular localization in immunofluorescence.

Thirdly, YCharOS experiments are not performed in replicates primarily due to the use of multiple antibodies targeting various epitopes. Once a specific antibody is identified, it validates the protein expression of the intended target in the selected cell line, confirms the lack of protein expression in the KO cell line and supports conclusions regarding the specificity of the other antibodies. All experiments are performed using master mixes, and meticulous attention is paid to sample preparation and experimental execution. In IF, the use of two different concentrations serves to evaluate antibody specificity and can aid in assessing assay reliability. In instances where antibodies yield no signal, a repeat experiment is conducted following titration. Additionally, our independent data is performed subsequently to the antibody manufacturers internal validation process, therefore making our characterization process a repeat.

Lastly, as comprehensive and standardized procedures are respected, any conclusions remain confined to the experimental conditions and cell line used for this study. The use of a single cell type for evaluating antibody performance poses as a limitation, as factors such as target protein abundance significantly impact results. ^7^ Additionally, the use of cancer cell lines containing gene mutations poses a potential challenge, as these mutations may be within the epitope coding sequence or other regions of the gene responsible for the intended target. Such alterations can impact the binding affinity of antibodies. This represents an inherent limitation of any approach that employs cancer cell lines.

## Method

The standardized protocols used to carry out this KO cell line-based antibody characterization platform was established and approved by a collaborative group of academics, industry researchers and antibody manufacturers. The detailed materials and step-by-step protocols used to characterize antibodies in western blot, immunoprecipitation and immunofluorescence are openly available on Protocol Exchange, a preprint server (DOI: 10.21203/rs.3.pex-2607/v1). ^7^ Brief descriptions of the experimental setup used to carry out this study can be found below.

### Cell lines and antibodies

Cell lines used and primary antibodies tested in this study are listed in Tables 1 and 2, respectively. To ensure that the cell lines and antibodies are cited properly and can be easily identified, we have included their corresponding Research Resource Identifiers, or RRID. ^11, 12^ HeLa *VAPB* KO clone was generated at the Early Drug Discovery Unit (EDDU) using a guide RNA with the following sequence: UGAAGACUACAGCACCACGU.

**Table 2.** Summary of the VAPB antibodies tested.

Company	Catalog number	Lot number	RRID (Antibody Registry)	Clonality	Clone ID	Host	Concentration (μg/μL)	Vendors recommended applications
Abcam	ab315013 **	1075876-3	AB_3086770	recombinant-mono	EPR27026-50	rabbit	0.51	Wb, IP
ABclonal	A5363	12860201	AB_2766173	polyclonal	-	rabbit	1.41	Wb, IF
GeneTex	GTX131631	43103	AB_2886511	polyclonal		rabbit	0.47	Wb, IP, IF
Proteintech	66191-1-Ig *	10004068	AB_2881586	monoclonal	4F6A6	mouse	1.40	Wb, IF
Thermo Fisher Scientific	MA5-29639 **	WA3152374	AB_2785468	recombinant-mono	208	rabbit	1.00	Wb
Thermo Fisher Scientific	PA5-53023	YF3969135A	AB_2649391	polyclonal	-	rabbit	0.30	Wb, IP, IF

Wb = western blot; IF = immunofluorescence; IP = immunoprecipitation.

* Monoclonal antibody.

** Recombinant antibody.

**Table 3.** Illustrations to assess antibody performance in all western blot, immunoprecipitation and immunofluorescence.

Western blot	Immunoprecipitation	Immunofluorescence
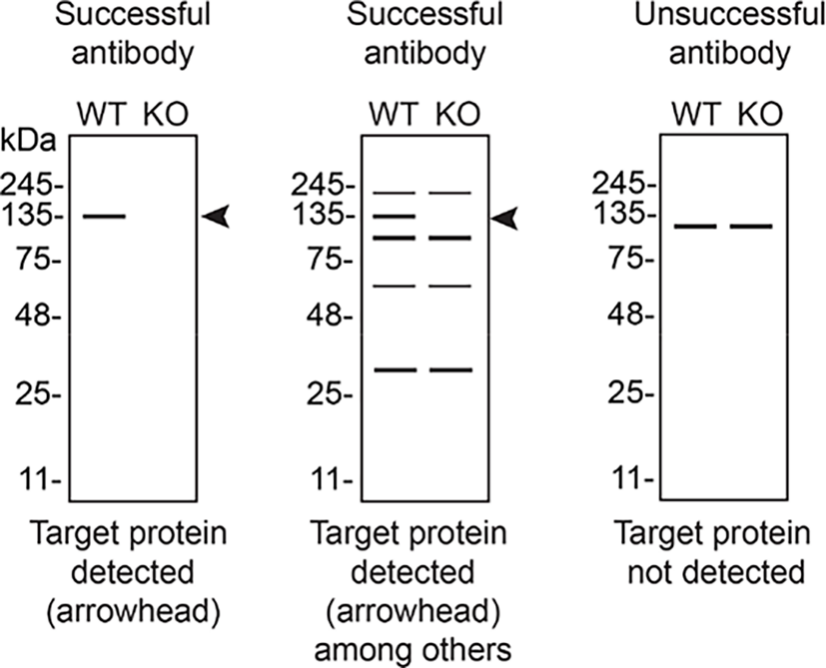	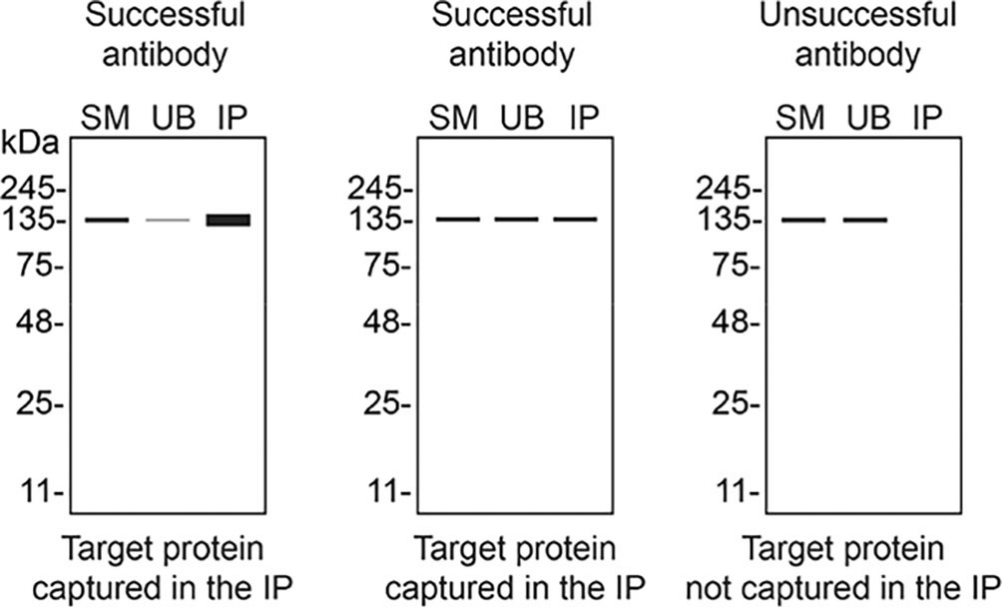	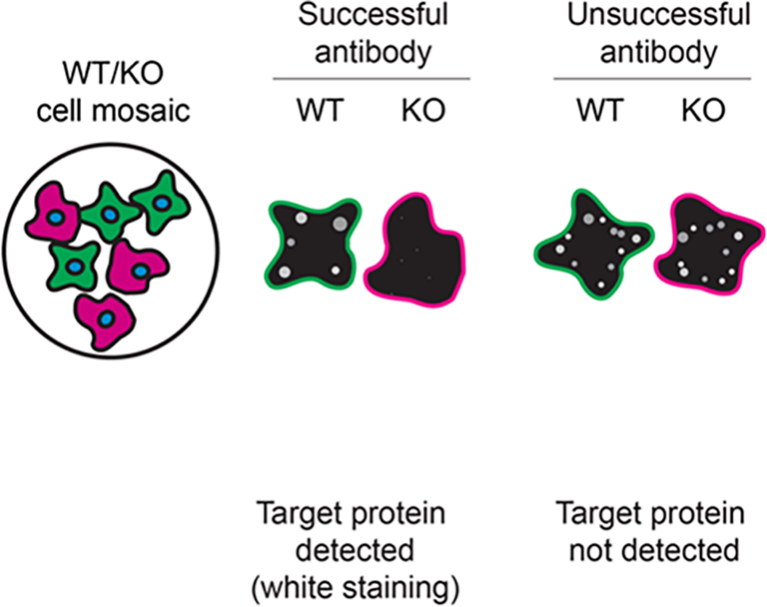

This table has been reproduced with permission from Ayoubi et al., Elife, 2023. ^4^

Peroxidase-conjugated goat anti-rabbit and anti-mouse antibodies are (Thermo Fisher Scientific, cat. number 65-6120 and 62-6520). Peroxidase-conjugated VeriBlot for IP detection is from Abcam, cat. number ab131366. Alexa-555-conjugated goat anti-rabbit and anti-mouse secondary antibodies (Thermo Fisher Scientific, cat. number A21429 and A21424).

### Antibody screening by western blot

HeLa WT and *VAPB* KO cells were collected in RIPA buffer (25 mM Tris-HCl pH 7.6, 150 mM NaCl, 1% NP-40, 1% sodium deoxycholate, 0.1% SDS) (Thermo Fisher Scientific, cat. number 89901) supplemented with 1× protease inhibitor cocktail mix (MilliporeSigma, cat. number P8340). Lysates were sonicated briefly and incubated 30 min on ice. Lysates were spun at ~110,000 × *g* for 15 min at 4°C and equal protein aliquots of the supernatants were analyzed by SDS-PAGE and western blot. BLUelf prestained protein ladder (GeneDireX, cat. number PM008-0500) was used.

Western blots were performed with precast midi 4-20% Tris-Glycine polyacrylamide gels (Thermo Fisher Scientific, cat. number WXP42012BOX) ran with Tris/Glycine/SDS buffer (Bio-Rad, cat. number 1610772), loaded in Laemmli loading sample buffer (Thermo Fisher Scientific, cat. number AAJ61337AD) and transferred on nitrocellulose membranes. Proteins on the blots were visualized with Ponceau S staining (Thermo Fisher Scientific, cat. number BP103-10) which is scanned to show together with individual western blot. Blots were blocked with 5% milk for 1 hr, and antibodies were incubated O/N at 4°C with 5% milk in TBS with 0.1% Tween 20 (TBST) (Cell Signalling Technology, cat. number 9997). Following three washes with TBST, the peroxidase conjugated secondary antibody was incubated at a dilution of ~0.2 μg/ml in TBST with 5% milk for 1 hr at room temperature followed by three washes with TBST. Membranes were incubated with Pierce ECL (Thermo Fisher Scientific, cat. number 32106) prior to detection with the iBright™ CL1500 Imaging System (Thermo Fisher Scientific, cat. number A44240).

### Antibody screening by immunoprecipitation

Antibody-bead conjugates were prepared by adding 2 μg of antibody to 500 μl of Pierce IP Lysis Buffer from Thermo Fisher Scientific (cat. number 87788) in a microcentrifuge tube, together with 30 μl of Dynabeads protein A- (for rabbit antibodies) or protein G- (for mouse antibodies) (Thermo Fisher Scientific, cat. number 10002D and 10004D, respectively). Tubes were rocked for ~1 h at 4°C followed by two washes to remove unbound antibodies.

HeLa WT were collected in Pierce IP buffer (25 mM Tris-HCl pH 7.4, 150 mM NaCl, 1 mM EDTA, 1% NP-40 and 5% glycerol) supplemented with protease inhibitor. Lysates were rocked 30 min at 4°C and spun at 110,000 × *g* for 15 min at 4°C. 0.5 ml aliquots at 2 mg/ml of lysate were incubated with an antibody-bead conjugate for ~1 h at 4°C. The unbound fractions were collected, and beads were subsequently washed three times with 1.0 ml of IP buffer and processed for SDS-PAGE and western blot on a precast midi 4-20% Tris-Glycine polyacrylamide gels. VeriBlot for IP Detection Reagent:HRP was used as a secondary detection system at a concentration of 0.1 μg/ml.

### Antibody screening by immunofluorescence

HeLa WT and *VAPB* KO cells were labelled with a green and a far-red fluorescence dye, respectively (Thermo Fisher Scientific, cat. number C2925 and C34565). The nuclei were labelled with DAPI (Thermo Fisher Scientific, cat. Number D3571) fluorescent stain. WT and KO cells were plated on 96-well plate with optically clear flat-bottom (Perkin Elmer, cat. number 6055300) as a mosaic and incubated for 24 hrs in a cell culture incubator at 37 ^o^C, 5% CO _2_. Cells were fixed in 4% paraformaldehyde (PFA) (VWR, cat. number 100503-917) in phosphate buffered saline (PBS) (Wisent, cat. number 311-010-CL). Cells were permeabilized in PBS with 0.1% Triton X-100 (Thermo Fisher Scientific, cat. number BP151-500) for 10 min at room temperature and blocked with PBS with 5% BSA, 5% goat serum (Gibco, cat. number 16210-064) and 0.01% Triton X-100 for 30 min at room temperature. Cells were incubated with IF buffer (PBS, 5% BSA, 0.01% Triton X-100) containing the primary VAPB antibodies overnight at 4°C. Cells were then washed 3 × 10 min with IF buffer and incubated with corresponding Alexa Fluor 555-conjugated secondary antibodies in IF buffer at a dilution of 1.0 μg/ml for 1 hr at room temperature with DAPI. Cells were washed 3 × 10 min with IF buffer and once with PBS.

Images were acquired on an ImageXpress micro confocal high-content microscopy system (Molecular Devices), using a 20x NA 0.95 water immersion objective and scientific CMOS cameras, equipped with 395, 475, 555 and 635 nm solid state LED lights (lumencor Aura III light engine) and bandpass filters to excite DAPI, Cellmask Green, Alexa-555 and Cellmask Red, respectively. Images had pixel sizes of 0.68 × 0.68 microns, and a z-interval of 4 microns. For analysis and visualization, shading correction (shade only) was carried out for all images. Then, maximum intensity projections were generated using 3 z-slices. Segmentation was carried out separately on maximum intensity projections of Cellmask channels using CellPose 1.0, and masks were used to generate outlines and for intensity quantification. ^13^ Figures were assembled with Adobe Illustrator.

## Data Availability

Zenodo: Antibody Characterization Report for VAPB, https://doi.org/10.5281/zenodo.13891570. ^14^ Zenodo: Dataset for the VAPB antibody screening study, https://doi.org/10.5281/zenodo.14503380. ^15^ Data are available under the terms of the Creative Commons Attribution 4.0 International license (CC-BY 4.0).
