# Supplementary material for: A guide to selecting high-performing antibodies for VAPB (UniProt ID: O95292) for use in western blot, immunoprecipitation, and immunofluorescence
Source: F1000Res. 2024 Dec 24;13:1559. [Version 1] doi: 10.12688/f1000research.160226.1 (PMC11799760; doi:10.12688/f1000research.160226.1)
Supplement: Peer review history

# Reviewer report: Reviewer response for version 1

**DOI:** 10.5256/f1000research.176085.r359974

**Published:** 2025-02-05

**Authors:** Jieya Shao

**Affiliations:** 1 Washington University in St Louis, St. Louis, Missouri, USA

In this article the authors systematically compared the performance and specificity of six commercial anti-VAPB antibodies using three different experimental approaches (Western blot, immunoprecipitation, and immunofluorescence staining). This study is part of a large collaborative initiative to characterize commercial antibodies for different human proteins and offer unbiased guidance to researchers who can select the best antibodies for their specific experimental objectives based on their own interpretations. As a Data Note, this paper successfully achieved its goal with rigorously collected high-quality data, and the results are self-explanatory and useful for the research community.

 No issues were found except a small grammatical error on page 3 under Results and discussion. The authors wrote "WT and VAPB KO protein lysates were ran on SDS PAGE". The correct word is "run".

Are sufficient details of methods and materials provided to allow replication by others?

Yes

Is the rationale for creating the dataset(s) clearly described?

Yes

Are the datasets clearly presented in a useable and accessible format?

Yes

Are the protocols appropriate and is the work technically sound?

Yes

Reviewer Expertise:

DNA replication, cancer biology

I confirm that I have read this submission and believe that I have an appropriate level of expertise to confirm that it is of an acceptable scientific standard.

# Reviewer report: Reviewer response for version 1

**DOI:** 10.5256/f1000research.176085.r359968

**Published:** 2025-02-05

**Authors:** Karen Bowman

**Affiliations:** 1 University of Leicester, Leicester, England, UK

The study involved characterisation of six commercial antibodies, from five different suppliers, for the vesicle-associated membrane protein-associated protein B/C (VAPB) for use in western blot, immunoprecipitation, and immunofluorescence. The study formed part of the wider YCharOS collaboration to characterise commercial antibodies for human proteins using standardised protocols with the intention of improving antibody reproducibility issues. As a Data Note, it allows the direct comparison of the performance of commercially available antibodies to VAPB protein to aid scientists in choosing the most appropriate antibody for their studies, for example, in the biochemical and cellular assessment of VAPB properties and function. This comparison would be of particular use in the field of neurodegenerative diseases, especially in the study of amyotrophic lateral sclerosis (ALS).

 A committee of industry and academic representatives have endorsed the platform used, and the protocols used are consistent with those in general use. The platform consisted of identification of a human cell line suitable for antibody characterisation studies i.e., with adequate levels of the VAPB protein to generate a measurable signal. They found that the commercially available HeLa WT cell line expressed VAPB transcript at a level above the average range of cancer cells analysed, and therefore, was a logical choice for their study. The matched isogenic knockout control cell line, HeLa *VAPB* KO was used as an appropriate negative control. The final step was a series of antibody characterisation procedures, limited to the most common research uses of antibodies. The interpretation of the results is left to the reader, with the study providing unbiased guidance. However, it gave some indication of the optimum dilutions of commercial antibodies. Limitations of the study are clearly stated. The summary Table 3 with illustrations to assess antibody performance in western blot, immunoprecipitation and immunofluorescence, is very helpful.

 I think this study could have been improved by increasing the number of commercially available antibodies studied. There are at least a further seven commercial VAPB antibodies available. However, the study is a useful starting point when choosing the most appropriate antibody for study of the VAPB protein.

Are sufficient details of methods and materials provided to allow replication by others?

Yes

Is the rationale for creating the dataset(s) clearly described?

Yes

Are the datasets clearly presented in a useable and accessible format?

Yes

Are the protocols appropriate and is the work technically sound?

Yes

Reviewer Expertise:

cell biology

I confirm that I have read this submission and believe that I have an appropriate level of expertise to confirm that it is of an acceptable scientific standard.

# Reviewer report: Reviewer response for version 1

**DOI:** 10.5256/f1000research.176085.r359970

**Published:** 2025-01-24

**Authors:** Sheng-ce Tao

**Affiliations:** 1 Shanghai Jiao Tong University, Shanghai, China

Because of the importance of VAPB, high quality antibody is a needed. This study evaluates six commercial antibodies against VAPB for western blot, immunoprecipitation, and immunofluorescence using standardized protocols in knockout and isogenic control cell lines. The research aims to address antibody reproducibility and provide open data to help researchers select the best antibodies for their studies.

 1. What are the criteria for selecting the six commercial antibodies? Please clarify.

 2. This study only tested one cell line (HeLa). It is not necessary that the conclusion from one cell generally also works among other cell lines. 

 3. Since one of the major application of these validated antibodies is WB, it is highly possible that these antibodies recognize linear epitopes on VAPB. In this case, mapping the epitopes (for example, using AbMap technology), and then judge the specificity by epitope searching across the whole proteome will be a more general  approach.

Are sufficient details of methods and materials provided to allow replication by others?

Yes

Is the rationale for creating the dataset(s) clearly described?

Yes

Are the datasets clearly presented in a useable and accessible format?

Yes

Are the protocols appropriate and is the work technically sound?

Yes

Reviewer Expertise:

Antibody technology, proteomics

I confirm that I have read this submission and believe that I have an appropriate level of expertise to confirm that it is of an acceptable scientific standard.

# Response

**Published:** 2025-01-30

**Authors:** Carl Laflamme

**Affiliations:** McGill University, Canada

Dear Dr. Sheng-ce Tao,

 Thank you for your review and feedback on our manuscript.

 In response to your points:

1. The YCharOS initiative collaborates with 12 antibody manufacturers, representing approximately 80% of all renewable antibodies commercially available. These partners have selected and provided their VAPB antibodies with us. We focus on renewable antibodies, as the YCharOS project does not undertake antibody characterization studies for proteins covered solely by polyclonal antibodies. The YCharOS workflow was recently featured and detailed in a News and Views article ( https://doi.org/10.1038/s41596-024-01108-6).
2. We acknowledge the reviewer's concern that a limitation of this study is the screening of antibodies in a single cell line. Our study serves as an antibody selection guide, but it remains essential for researchers to validate antibody specificity and performance within their own experimental context. As stated in the limitations section: “ *Lastly, as comprehensive and standardized procedures are respected, any conclusions remain confined to the experimental conditions and cell line used for this study. The use of a single cell type for evaluating antibody performance poses as a limitation, as factors such as target protein abundance significantly impact results (8). Additionally, the use of cancer cell lines containing gene mutations poses a potential challenge, as these mutations may be within the epitope coding sequence or other regions of the gene responsible for the intended target. Such alterations can impact the binding affinity of antibodies. This represents an inherent limitation of any approach that employs cancer cell lines*”.
3. Mapping the epitopes of the tested antibodies falls outside the scope of the YCharOS initiative.

Sincerely

 Carl Laflamme

# Reviewer report: Reviewer response for version 1

**DOI:** 10.5256/f1000research.176085.r357897

**Published:** 2025-01-15

**Authors:** Mórotz Gábor Miklós

**Affiliations:** 1 Semmelweis University, Budapest, Budapest, Hungary

In this paper Gonzales Bolivar and colleagues tested six commercially available anti-VAPB antibodies as part of the YCharOS initiative. The experimental results are good quality and clearly presented. These data allow fellow researchers to directly compare the performance of the selected antibodies side-by-side. I have only a few comments.

 1. In the introduction section, please use more appropriate references for the cellular function of VAPB (e.g. Dudás EF, et al., 2021 [Ref 1]). Additionally, the major cellular functions regulated by VAPB tethering are lipid metabolism and calcium homeostasis.

 2. It is not clear for me what were the exact antibody selection criteria for the study. For example, I can see monoclonal, recombinant and polyclonal antibodies on the list so clearly this was not a selection criterion although in the Limitations section is written that “project focuses on renewable (recombinant and monoclonal) antibodies”. It would be nice to add a flow-chart summarising how many antibodies are on the market and based on what criteria were these six antibodies selected for the work. In this way, the reader can decide how comprehensive this study is.

 3. VAPB is an integral ER membrane protein. Therefore, for the immunofluorescent experiments it would be very informative to use an ER staining instead of a general cell staining to analyse whether the tested antibodies recognise the protein in the ER.

 4. In the Results and discussion section first paragraph please correct “HeLa expresses the VAPB” to HeLa cells express the VAPB.

 5. In the Methods section please correct the “peroxidase-conjugated antibodies” to horseradish peroxidase-conjugated antibodies. I also suggest using h instead of hr or hrs for the abbreviation of hour as it is more widely used in the scientific literature.

Are sufficient details of methods and materials provided to allow replication by others?

Yes

Is the rationale for creating the dataset(s) clearly described?

Yes

Are the datasets clearly presented in a useable and accessible format?

Yes

Are the protocols appropriate and is the work technically sound?

Yes

Reviewer Expertise:

neurodegeneration, VAPB

I confirm that I have read this submission and believe that I have an appropriate level of expertise to confirm that it is of an acceptable scientific standard, however I have significant reservations, as outlined above.

## References

[1] : Invisible leashes: The tethering VAPs from infectious diseases to neurodegeneration. *J Biol Chem* .2021;296: 10.1016/j.jbc.2021.100421 100421 10.1016/j.jbc.2021.100421 33609524 PMC8005810
